# Spin-lattice decoupling in a triangular-lattice quantum spin liquid

**DOI:** 10.1038/s41467-018-04005-1

**Published:** 2018-04-17

**Authors:** Takayuki Isono, Shiori Sugiura, Taichi Terashima, Kazuya Miyagawa, Kazushi Kanoda, Shinya Uji

**Affiliations:** 1 National Institute for Materials Science, Tsukuba, Ibaraki 305-0003 Japan; 2 Department of Applied Physics, University of Tokyo, Bunkyo-ku, Tokyo, 113-8656 Japan; 3 Present Address: Condensed Molecular Materials Laboratory, RIKEN, Wako, Saitama, 351-0198 Japan

## Abstract

A quantum spin liquid (QSL) is an exotic state of matter in condensed-matter systems, where the electron spins are strongly correlated, but conventional magnetic orders are suppressed down to zero temperature because of strong quantum fluctuations. One of the most prominent features of a QSL is the presence of fractionalized spin excitations, called spinons. Despite extensive studies, the nature of the spinons is still highly controversial. Here we report magnetocaloric-effect measurements on an organic spin-1/2 triangular-lattice antiferromagnet, showing that electron spins are decoupled from a lattice in a QSL state. The decoupling phenomena support the gapless nature of spin excitations. We further find that as a magnetic field is applied away from a quantum critical point, the number of spin states that interact with lattice vibrations is strongly reduced, leading to weak spin–lattice coupling. The results are compared with a model of a strongly correlated QSL near a quantum critical point.

## Abstract (web summary)

A number of materials have been proposed as realizations of exotic quantum spin liquids but many important properties are difficult to establish. Isono et al. show evidence for spin-lattice decoupling in an organic material, which may help resolve conflicting results about the existence of a spin-excitation gap.

## Introduction

A quantum spin liquid (QSL) is an intriguing exception for the Landau theory of phase transitions; at sufficiently low temperatures, condensed-matter systems form an ordered state characterized by broken symmetries and corresponding order parameters. However, the order can be suppressed when there exist strong quantum-mechanical fluctuations enhanced by low dimensionality and/or geometrical frustration. The resulting exotic quantum liquids such as the QSL are not described by any broken symmetry or order parameter^1^. The QSL is also of great interest in connection with a mechanism of high-temperature superconductivity^2^ and application to quantum computation^3^.

A spin-1/2 triangular-lattice antiferromagnet with nearest-neighbor (NN) antiferromagnetic (AF) exchange interactions *J* is one of the most typical example of two-dimensional frustrated spin systems, in which Anderson first proposed a QSL ground state more than 40 years ago^4^. Although the ground state of the triangular-lattice Heisenberg AF system is now known to be 120° AF order^5^, its ordered state can be suppressed by ring-exchange interactions^6^, next NN interactions^7^, or a spatial distribution of an exchange coupling constant^8^, consequently leading to QSL ground states. One of the most fundamental properties of a QSL is the presence of charge neutral excitations carrying spin-1/2 quantum number, spinons. These fractional excitations are clearly distinct from spin-1 magnon excitations in magnetically ordered states. Depending on the theoretical model, the spinon excitations may be gapped or gapless, and may obey Bose or Fermi statistics^1,6,9–11^.

In 2003, the first evidence of a QSL was reported in an organic triangular-lattice antiferromagnet, *κ*-(BEDT-TTF)_2_Cu_2_(CN)_3_ (ref. ^12^), where BEDT-TTF stands for bis(ethylenedithio)tetrathiafulvalene. In this material, a spin-1/2 is located on a (BEDT-TTF)$_2^ +$ dimer, which is arranged on a triangular lattice. Despite the large NN AF interactions, *J*/*k*_B_ ~ 250 K, no magnetic long-range order happens down to *T ∼ *30 mK^12–14^, which is four orders of magnitude lower than *J*/*k*_B_. This suggests that the QSL state is realized in *κ*-(BEDT-TTF)_2_Cu_2_(CN)_3_. The nature of the magnetic excitations of the QSL state, spinons, has been intensively studied and discussed. A finite value of the specific heat divided by temperature *C*/*T* for *T* → 0, and Pauli-like magnetic susceptibility are hallmarks of gapless spin excitations^15,16^. Conversely, Arrhenius behavior of the thermal conductivity suggests the presence of a small gap, Δ/*k*_B_ ~ 0.5 K^17^. There have been many debates about the spinon excitations not only in *κ*-(BEDT-TTF)_2_Cu_2_(CN)_3_, but also in the other QSL candidates such as a triangular-lattice system, YbMgGaO_4_^18,19^, and a kagome-lattice system, ZnCu_3_(OH)_6_Cl_2_ (refs. ^20,21^).

Here we report magnetocaloric-effect (MCE) measurements on *κ*-(BEDT-TTF)_2_Cu_2_(CN)_3_, which unveils a characteristic thermal relaxation of the QSL state. At very low temperatures in a magnetic field, the thermal relaxation time between the electron spins and lattice is dramatically increased, indicating that the spins are decoupled from the lattice bath. The spin–lattice decoupling can explain the seeming discrepancy in the nature of the spin-excitation spectrum; the spin excitations are gapless. Moreover, we show by combining the present MCE results with our recent magnetic-susceptibility study that as a zero-field quantum critical point (QCP) is approached, the number of spin states is strongly enhanced. This is compatible with a model of a strongly correlated QSL near a QCP.

## Results

### Magnetocaloric effect

**Fig. 1:**
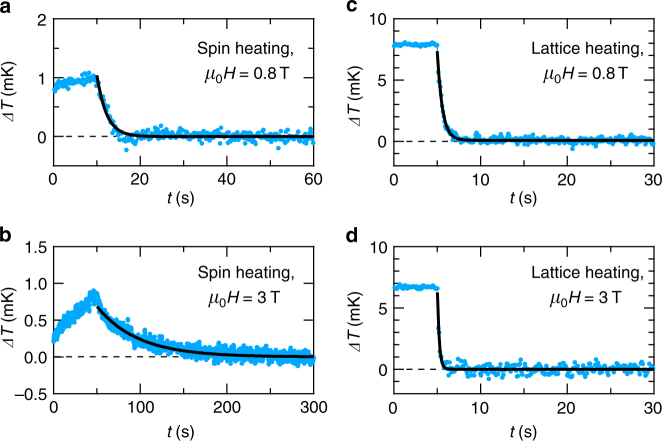
Thermal relaxation curve at the temperature of 0.26 K. Thermal relaxation curve for the spin (magnetocaloric) heating, when the magnetic field is swept up from **a** 0.6 to 0.8 T, and **b** 2 to 3 T. The thermal relaxation curve for the lattice (Joule) heating at **c** *μ*_0_*H* = 0.8 T and **d** 3 T. In each figure, the solid line represents a single exponential decay, Δ*T* = *A* exp(−*t*/*τ*), with the relaxation time, *τ*, and a constant, *A*

In the present study, we have measured the MCE to resolve the discrepancy between the gapped and gapless features of the spin excitations in *κ*-(BEDT-TTF)_2_Cu_2_(CN)_3_. The MCE, Δ*T*, is a thermal response of a sample to magnetic-field changes, d*H*/d*t*, given by

1 $${\mathrm{\Delta }}T = - \tau \frac{{{\mathrm{d}}({\mathrm{\Delta }}T)}}{{{\mathrm{d}}t}} - \frac{T}{{K_{\mathrm{B}}}}\frac{{{\mathrm{d}}H}}{{{\mathrm{d}}t}}\left( {\frac{{\partial S}}{{\partial H}}} \right)_T,$$

where *K*_B_ represents the thermal conductance between the sample and heat bath. The second term in Eq. (1) describes heating or cooling of the sample by the magnetic entropy change, d*S*/d*H*. When *S* decreases (increases) with increasing field, the sample is heated up (cooled down). Resulting Δ*T* is relaxed with the relaxation time, *τ* = *C*/*K*_B_, as described in the first term. Here, *C* represents the heat capacity of the sample. Figure 1a, b show heating and relaxation processes of the sample at the bath temperature, *T*_B_, of 0.26 K (see also Supplementary Fig. 1). As the field increases up to 0.8 T (*t* < 10 s) (Fig. 1a), the sample temperature increases, Δ*T* > 0 K. We observe positive Δ*T* in the whole field and temperature range up to 17 T between 0.1 and 1.6 K, indicating that the magnetic entropy is monotonously suppressed by applying a magnetic field. This is a clear contrast to a quantum disordered state with a singlet–triplet gap being closed in high fields, where the increase of the entropy results in negative Δ*T*^22,23^. Hereafter, we call the heating by the MCE the spin heating. At *μ*_0_*H* = 0.8 T (*t* = 10 s), the field is stopped [d*H*/d*t* = 0 in Eq. (1)] and then we observe the relaxation of Δ*T*, which is well represented as a single exponential decay, Δ*T* ~ exp(−*t*/*τ*), with relaxation time *τ* = 2.2 s (solid line). By applying the magnetic field of 3 T, *τ* dramatically increases to 47 s (Fig. 1b). By contrast, when the sample is heated by an electric heater (called the lattice heating below), *τ* remains very short in the whole field region, as shown in Fig. 1c, d: *τ* = 0.5 and 0.2 s at *μ*_0_*H* = 0.8 and 3 T, respectively. Here, we note that *τ* ~ 2 s for the spin heating is attributed to the time constant of a superconducting magnet used in the present measurements, as shown in Supplementary Fig. 2; actual *τ* is shorter than 2 s.

**Fig. 2:**
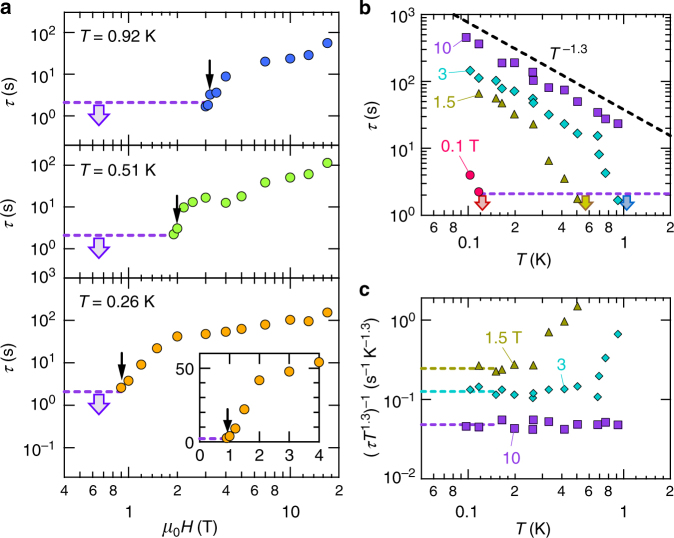
Magnetic field and temperature dependence of the relaxation time. **a** Magnetic-field dependence of the relaxation time, *τ*, for the spin heating at various temperatures. The dashed line represents constant *τ* of 2.1 s. At low fields, *τ* is shorter than 2.1 s (thick arrows). The thin arrows indicate the onset field of the *τ* increase, H*, which is determined based on the linear-scale plot, shown in the inset. **b** Temperature variation in *τ* for the spin heating at various magnetic fields. The black- and purple-dashed lines denote the power-law behavior with an exponent of −1.3, and constant *τ* of 2.1 s, respectively. At each field, *τ* is shorter than 2.1 s above the temperature indicated by the thick arrows. **c** Temperature dependence of (*τT*^1.3^)^−1^ at various magnetic fields. The dashed line represents a zero-temperature extrapolation of the low-temperature data, $(\tau T^{1.3})_0^{ - 1}$

The magnetic-field dependence of the relaxation time for the spin heating is summarized in Fig. 2a. At *T*_B_ = 0.26 K, *τ* is shorter than 2 s up to *μ*_0_*H* ~ 0.9 T. Above this field, *τ* exceeds 2 s and rapidly increases more than one order of magnitude, followed by a slow increase without showing saturation behavior above ~2 T. Here, we determine the onset field of the *τ* increase, H*, based on the linear-scale plot of *τ*(*H*) (inset of Fig. 2a). With temperature elevation, H* monotonically shifts to a higher value. Figure 2b depicts the relaxation time as a function of temperature. At *μ*_0_*H* = 0.1 T, *τ* is shorter than 2 s down to *T*_B_ ~ 0.12 K, below which a rapid increase of *τ* is observed. The onset temperature of the *τ* increase is raised by applying a magnetic field. At 1.5 T, *τ* is rapidly increased below 0.5 K, and after that *τ* shows a rather slow increase. At 10 T, we observe power-law behavior, *τ* ~ *T*^−1.3^, in the wide temperature range. Figure 2c shows the temperature dependence of (*τT*^1.3^)^−1^. As the temperature goes to zero, (*τT*^1.3^)^−1^ approaches a constant value (dashed lines). This value is monotonically decreased as a magnetic field is increased.

### Simulation results

**Fig. 3:**
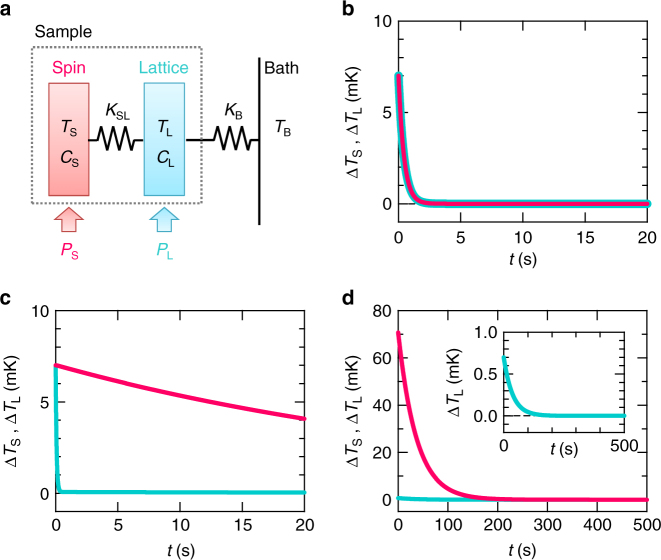
Simulation results of the thermal relaxation. **a** Schematic diagram of the experimental situation. The heat supplied to the spin system first flows to the lattice system through the thermal conductance, *K*_SL_, and then to the heat bath through *K*_B_. *P*_S_ (*P*_L_) and *C*_S_ (*C*_L_) represent the heating power of the spin (lattice) heating and the heat capacity of the spin (lattice) system, respectively. *T*_S_, *T*_L_, and *T*_B_ denote the temperature of the spins, lattice, and bath, respectively. **b** The thermal relaxation curve when the spins are strongly coupled to the lattice [Δ*T*_S_(*t*) = *T*_S_(*t*) − *T*_B_ and Δ*T*_L_(*t*) = *T*_L_(*t*) − *T*_B_]. The thermal relaxation curve when the spins are decoupled from the lattice for **c** the lattice heating and **d** the spin heating. Inset of **d**: the enlarged figure of the main panel. Magenta and cyan lines represent Δ*T*_S_(*t*) and Δ*T*_L_(*t*), respectively

An important question here is why the slow relaxation takes place only for the spin heating. To address this question, we simulate the thermal relaxation curve for the spin and lattice heatings, based on a simple spin–lattice coupling model, as shown in Fig. 3a (details of the simulations are shown in Methods). Our MCE measurements probe the temperature of the lattice system, *T*_L_. In a conventional case, the thermal coupling between the spins and lattice, *K*_SL_, is sufficiently stronger than lattice–bath coupling, *K*_B_: *K*_SL_ $\gg$ *K*_B_. In this case, the temperature of the spin system, *T*_S_, is identical to *T*_L_ for both the spin and lattice heatings (*P*_S_ and *P*_L_, respectively), as shown in Fig. 3b, Supplementary Fig. 3(a), and its inset. The relaxation time is given by *τ* = (*C*_S_ + *C*_L_)/*K*_B_ = 0.4 s, which is confirmed by the exponential decay fit to the relaxation curve. Here, *C*_S_ and *C*_L_ represent the heat capacities of the spins and lattice, respectively. When the spins are decoupled from the lattice (*K*_SL_ $\ll$ *K*_B_), *T*_L_ is rapidly raised by the lattice heating, whereas *T*_S_ slowly increases because of small *K*_SL_ (Supplementary Fig. 3(b)). After stopping the heating, *T*_L_ is quickly relaxed, given by $\tau \sim C_{\mathrm{L}}{\mathrm{/}}K_{\mathrm{B}}$ = 0.1 s, as shown in Fig. 3c. In case of the spin heating, by contrast, supplied heat can hardly be relaxed to the lattice, and consequently, *T*_S_ becomes much higher than *T*_L_, as shown in Supplementary Fig. 3(c) and its inset. After stopping the heating, *T*_S_ and *T*_L_ are slowly relaxed to the bath temperature, *T*_B_. The relaxation time is given by $\tau \sim C_{\mathrm{S}}{\mathrm{/}}K_{{\mathrm{SL}}}$ = 40 s, which is much longer than that for the lattice heating (Fig. 3d and its inset). The above spin–lattice decoupling model reasonably explains the significant difference of *τ* between the spin and lattice heatings shown in Fig. 1b, d.

## Discussion

The spin–lattice decoupling requires reconsideration of the data analysis in the heat-capacity measurement on *κ*-(BEDT-TTF)_2_Cu_2_(CN)_3_. The heat capacity has been measured in a limited temperature and field region, *T* > 0.8 K and *μ*_0_*H* < 8 T^15^. In this region, *C* is obtained by measuring the thermal relaxation in a long time scale, 10–100 s. This time scale is much longer than our lattice-heating result, 0.1–1 s, and even comparable to the spin-heating one, <20 s (Fig. 2b). Therefore, *C*_S_ as well as *C*_L_ is measured in ref. ^15^, and consequently the finite *C*/*T* value for *T* → 0 is observed. An important implication of our MCE study is that the electronic spin entropy is monotonously decreased as a magnetic field is increased (${\mathrm{\Delta }}T$ ~ −d*S*/d*H* > 0). From the Maxwell’s relation (d*S*/d*H*)_*T*_ = (d*M*/d*T*)_*H*_, we obtain (d*M*/d*T*)_*H*_ < 0, where *M* represents the magnetization. This is consistent with the temperature dependence of the magnetic susceptibility below about 4 K and 3 T^16^. By contrast, no remarkable field dependence of the specific heat has been observed down to *T* ~ 0.8 K, suggesting d*S*/d*H* ~ 0^15^. The heat-capacity measurement in a magnetic field at lower temperatures, where the field dependence should become more pronounced, is highly required in the future.

**Fig. 4:**
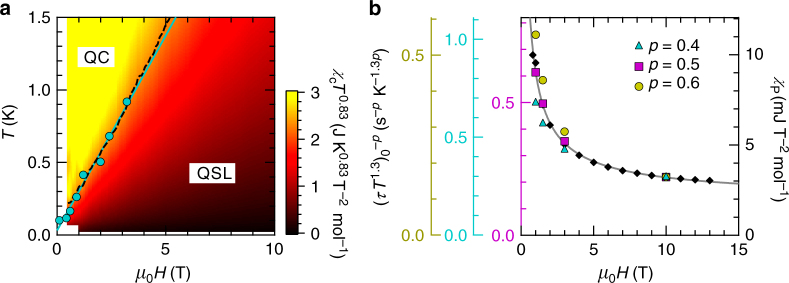
Comparison of the MCE results with the susceptibility data. **a** Contour plot of the magnetic susceptibility multiplied by the power of temperature, *χT*^0.83^, in the *T*–*H* plane, determined by us in ref. ^16^. The yellow and dark-red areas represent the quantum critical (QC) and QSL states, respectively, which are separated by the crossover region (light-red area). The onset field of the *τ* increase, H*, shown by the circles, falls on the contour line (dashed line), *χT*^0.83^ = 2.3 mJ K^0.83^ T^−2^ mol^−1^. The blue solid line indicates a linear fit of H*. **b** The zero-temperature extrapolation of the (*τT*^1.3^)^−1^ values raised to the power *p*, $(\tau T^{1.3})_0^{ - p}$, and the Pauli-like susceptibility, *χ*_P_, as a function of magnetic field. *χ*_P_ is determined based on the susceptibility data in ref. ^16^. The solid line represents a power-law fit of *χ*_P_ with the exponent −0.73

Not only the heat capacity, but also the heat-transport measurement is affected by the spin–lattice decoupling. It has been reported that in cuprate superconductors, a poor contact between electrons and a lattice prevents a heat transfer between them, which leads to the strong suppression of the electronic thermal conductivity^24^. Likewise, once the spins are decoupled from the lattice, the spin contribution to the thermal conductivity will be significantly reduced. This decoupling is likely the origin of the rapid decrease of *κ*/*T* at very low temperatures for *κ*-(BEDT-TTF)_2_Cu_2_(CN)_3_^17^. On the other hand, *κ* is gradually increased by applying a magnetic field. A possible scenario is a model of a strongly correlated QSL (SCQSL) located near a fermion-condensation quantum-phase-transition point, where the QSL plays the role of heavy-fermion liquids placed into insulators^25^; as a magnetic field is increased away from the QCP, the effective mass of spinons, m*, is reduced, leading to the increase of the spin thermal conductivity. In fact, we have recently determined the *H*–*T* phase diagram based on a scaling analysis of *χ*, where a QCP is present near the zero field (Fig. 4a)^16^. In the quantum critical (QC) region (yellow area), *χ* diverges for *T* → 0, while *χ* shows almost *T*-independent (Pauli-paramagnetic-like) behavior in the QSL region (dark-red area). The Pauli-like susceptibility is commonly observed in organic triangular-lattice QSL materials^26,27^, and its values, *χ*_P_, are explained by a model of a QSL with a spinon Fermi surface^6,27^. In this model, fermionic spinons play the role of metallic electrons placed into insulators; *χ*_P_ comes from the spinon density of states at the Fermi level, *N*(*E*_F_), being proportional to m*. In this context, we examine the magnetic-field dependence of *χ*_P_ for *κ*-(BEDT-TTF)_2_Cu_2_(CN)_3_ in Fig. 4b, where the *χ*_P_ values are determined based on the susceptibility data reported by us^16^. By the application of a magnetic field, *χ*_P_ is decreased following a power law, $\chi _{\mathrm{P}} \sim H^{ - a}$, with the exponent *a* = 0.7–0.8, suggesting that as the system is away from the QCP, *N*(*E*_F_) ∝ m* is strongly suppressed. This is compatible with the SCQSL model.

In order to compare the MCE results with the susceptibility data, we plot H* on the *H*–*T* phase diagram, determined by us in ref.^16^ (Fig. 4a). A striking finding is that the data points of H* fall on the contour line, *χT*^0.83^ = 2.3 mJ K^0.83^ T^−2^ mol^−1^ (dashed line), in the crossover region (light-red area). This coincidence implies that low-energy spin excitations giving *χ*_P_ are also responsible for the thermal relaxation phenomena through coupling to lattice vibrations. In Fig. 4b, we further examine this relationship by comparing the field dependence of *χ*_P_ with the zero-temperature extrapolations of (*τT*^1.3^)^−1^(see Fig. 2c), raised to different powers, $(\tau T^{1.3})_0^{ - p}$. As the zero-field QCP is approached, $(\tau T^{1.3})_0^{ - p}$ with the exponent *p* = 0.5 is strongly increased in a manner similar to *χ*_P_. This suggests that $(\tau T^{1.3})_0^{ - 0.5}$ depends on *N*(*E*_F_) as well; the electron spin–lattice relaxation rate could be given by $\tau ^{ - 1} \sim [N(E_{\mathrm{F}})]^2T^{1.3}$ in the QSL state. The enhancement of *N*(*E*_F_) for *H* → 0 is intuitively consistent with the thermal-decoupling phenomena observed here; the application of a magnetic field reduces the number of spin states that interact with lattice vibrations, leading to the weak spin–lattice coupling.

In relation to the spin–lattice decoupling phenomena, several theoretical works have studied the interaction of spinons with phonons^28,29^. In a QSL with a spinon Fermi surface, spinons undergo pairing instability^30,31^, similar to the Cooper pairing in metals. The resulting gapped state reduces *N*(*E*_F_), leading to the weak spinon–phonon interactions^28^. However, this is not applicable to our case because no sign of a phase transition near H* has been found in thermodynamic quantities such as the specific heat and the magnetic torque^15,16^.

The main findings of the present MCE study are summarized in the following two points. First, we find the spin–lattice decoupling phenomena in the QSL state, which can explain the seeming discrepancy between the gapped^17^ and gapless^15,16^ features of spin excitations in *κ*-(BEDT-TTF)_2_Cu_2_(CN)_3_; spin excitations are gapless. Recently, the inorganic triangular-lattice antiferromagnet, YbMgGaO_4_, has been discussed as a candidate material for the QSL with the spinon Fermi surface. In this material, the gapless nature of spinons has been reported by the neutron-scattering and specific-heat experiments^18,19^, whereas the spin thermal conductivity appears to be absent^19^. The MCE study on YbMgGaO_4_ may resolve the discrepancy. Second, as the system is away from the QCP, the number of spin states is rapidly decreased, and consequently the spin–lattice interaction is weakened. This is compatible with the SCQSL model^25^, where the QSL has much similarity with heavy-fermion liquids.

## Methods

### Sample preparation and MCE measurements

Single-crystalline samples were prepared by electrochemical oxidation of BEDT-TTF molecules. In magnetocalorimetry, several single-crystalline samples of 227 μg were attached to a small thermometer (Cernox, Lake Shore) by a grease (Apiezon N Grease), and then the composition was enclosed in a home-made miniature vacuum cell, together with a reference thermometer. A temperature difference between the two thermometers, Δ*T*, was measured with a magnetic field swept up to 17 T at a sweep rate of 0.5 T min^−1^. All the measurements were made using a 20 T superconducting magnet with a dilution refrigerator at Tsukuba Magnet Laboratory, NIMS. A magnetic field is applied approximately perpendicular to a two-dimensional triangular-lattice plane.

### Thermal relaxation measurements and simulations

We have applied two heating methods for the thermal relaxation measurements, spin (magnetocaloric) and lattice (Joule) heatings. A simplified diagram of the experimental configuration is shown in Fig. 3a. Here, we assume that the lattice is strongly coupled to a thermometer; the ‘lattice’ in the figure includes addenda (a thermometer and grease). This condition is well satisfied when a thermometer is tightly attached to samples by a grease, as in the present case. Our relaxation measurements probe the lattice temperature, *T*_L_.

In the spin heating, spins are directly heated up by sweeping a magnetic field when d*S*/d*H* in Eq. (1) has a negative value. The heat supplied to the spins first flows to the lattice through the thermal conductance, *K*_SL_, and then to a heat bath through *K*_B_. After stopping the heating (field sweep), the spin temperature, *T*_S_, and *T*_L_ are relaxed to the bath temperature, *T*_B_. In the case of the lattice heating, the lattice is directly heated up by an electrical heater, while *T*_S_ is raised by a heat transfer from the lattice. The thermometer is also used as a heater in our experimental set-up. After stopping the heating, *T*_S_ and *T*_L_ are relaxed to *T*_B_.

In order to simulate the thermal relaxation curves for the two heating methods, we begin with heat balance equations for the above model (Fig. 3a),

2 $$P_{\mathrm{S}} = C_{\mathrm{S}}\frac{{{\mathrm{d}}T_{\mathrm{S}}}}{{{\mathrm{d}}t}} + K_{{\mathrm{SL}}}(T_{\mathrm{S}} - T_{\mathrm{L}}),$$

3 $$P_{\mathrm{L}} = C_{\mathrm{L}}\frac{{{\mathrm{d}}T_{\mathrm{L}}}}{{{\mathrm{d}}t}} + K_{{\mathrm{SL}}}(T_{\mathrm{L}} - T_{\mathrm{S}}) + K_{\mathrm{B}}(T_{\mathrm{L}} - T_{\mathrm{B}}).$$

Here, *P*_S_ (*P*_L_) and *C*_S_ (*C*_L_) represent the heating power of the spin (lattice) heating, and the heat capacity of the spins (lattice), respectively. By substituting d*T*/d*t* = [*T*(*t* + Δ*t*) − *T*(*t*)]/Δ*t* into Eqs. (2) and (3), we obtain

4 $$T_{\mathrm{S}}(t + {\mathrm{\Delta }}t) = T_{\mathrm{S}}(t) - \frac{{{\mathrm{\Delta }}t}}{{C_{\mathrm{S}}}}\{ K_{{\mathrm{SL}}}[T_{\mathrm{S}}(t) - T_{\mathrm{L}}(t)] - P_{\mathrm{S}}\},$$

5 $$\begin{array}{*{20}{l}}{T_{\mathrm{L}}(t + {{\mathrm{\Delta}}}t)} \hfill & = \hfill & T_{\mathrm{L}}(t) - \frac{{{\mathrm{\Delta}}t}}{{C_{\mathrm{L}}}}\left\{K_{{\mathrm{SL}}}\left[T_{\mathrm{L}}(t) - T_{\mathrm{S}}(t)\right]\right.\\ \hfill & {} \hfill & \left. + K_{\mathrm{B}}[T_{\mathrm{L}}(t) - T_{\mathrm{B}}(t)] - P_{\mathrm{L}}\right\}.\end{array}$$

Based on Eqs. (4) and (5), we simulate the thermal relaxation curve at *T*_B_ = 0.26 K and *μ*_0_*H* = 3 T. The spin and lattice contribution to the heat capacity of *κ*-(BEDT-TTF)_2_Cu_2_(CN)_3_ have been reported to be 730 and 90 pJ K^−1^ at *T* = 0.26 K, respectively^15^. The heat capacity of addenda (thermometer and grease) is about 60 pJ K^−1^, and then *C*_S_ = 730 pJ K^−1^ and *C*_L_ = 150 pJ K^−1^. When the sample is heated up by *P*_L_ = 14 pW, *T*_L_ is raised about 7 mK, as shown in Fig. 1d. These values together with a relation Δ*T*_L_ = *P*_L_/*K*_B_ give *K*_B_ = 2 nW K^−1^. Here, we consider two limiting conditions, conventional and decoupling cases. In the former case, the thermal coupling between the spins and lattice is sufficiently stronger than the lattice–bath coupling, *K*_SL_ $\gg$ *K*_B_. In this case, *T*_S_ is identical to *T*_L_ during the heating and relaxation processes, regardless of the heating methods (Supplementary Fig. 3(a), its inset, and Fig. 3(b)). The simulations are made using *K*_SL_ = 100 *K*_B_, *P*_L_ = 14 pW for the lattice heating, and *P*_S_ = 14 pW for the spin heating. The relaxation time is given by *τ* = (*C*_S_ + *C*_L_)/*K*_B_ = 0.4 s. In the latter case, the spins are decoupled from the lattice, *K*_SL_ $\ll$ *K*_B_. In case of the lattice heating, *T*_L_ is first raised, and after that *T*_S_ slowly increases owing to a small heat transfer from the lattice (Supplementary Fig. 3(b)). After stopping the heating, *T*_L_ is quickly relaxed, given by $\tau \sim C_{\mathrm{L}}{\mathrm{/}}K_{\mathrm{B}}$ = 0.1 s, as shown in Fig. 3c. The magnetic specific heat no longer contributes to the relaxation. In case of the spin heating, by contrast, the supplied heat is accumulated in the spins, because the heat can hardly flow to the lattice. Consequently, *T*_S_ becomes much higher than *T*_L_, as shown in Supplementary Fig. 3(c) and its inset. After stopping the heating, *T*_S_ and *T*_L_ are slowly relaxed, following $\tau \sim C_{\mathrm{S}}/K_{{\mathrm{SL}}}$ = 40 s (Fig. 3d and its inset), which is much longer than that for the lattice heating. The simulations are made using *K*_SL_ = *K*_B_/100, *P*_L_ = 14 pW for the lattice heating, and *P*_S_ = 1.4 pW for the spin heating. The spin–lattice decoupling model (Fig. 3c, d) reasonably explains why the slow thermal relaxation is observed only for the spin heating (Fig. 1b, d).

### Data availability

The data that support the findings of this study are available from the corresponding author on reasonable request.

## Electronic supplementary material

Supplementary Information
