# Supplementary material for: Spin-lattice decoupling in a triangular-lattice quantum spin liquid
Source: Nat Commun. 2018 Apr 17;9:1509. doi: 10.1038/s41467-018-04005-1 (PMC5904176; doi:10.1038/s41467-018-04005-1)
Supplement: Supplementary file 1 — Supplementary Information [file 41467_2018_4005_MOESM1_ESM.pdf]

Spin-lattice decoupling in a triangular-lattice quantum spin liquid

Isono et al.

Supplementary Figures

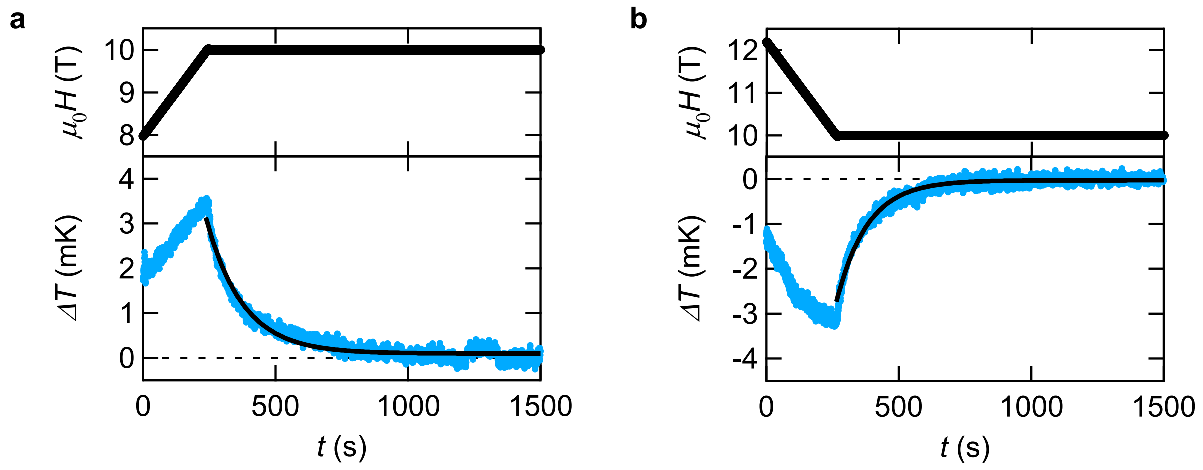

Supplementary Figure 1 | Heating and relaxation processes at T = 0.26 K and $\mu_0 H$ = 10 T. Time dependence of the temperature difference, ΔT, and the magnetic field, when the field is swept up (a) and down (b). Each solid line represents a single-exponential decay, $\Delta T \sim A\exp(-t/\tau)$ with relaxation time τ and a constant A. We must take care that at very low temperatures, changing the magnetic field induces an eddy current, which may heat up the vacuum cell. Such an extrinsic heating is absent in our measurements. The sign of ΔT is changed depending on the sweep direction of the magnetic field, as can be seen in Eq. (1) in the main text, which clearly shows that ΔT is generated by the magnetocaloric effect, but not by the eddy-current effect.

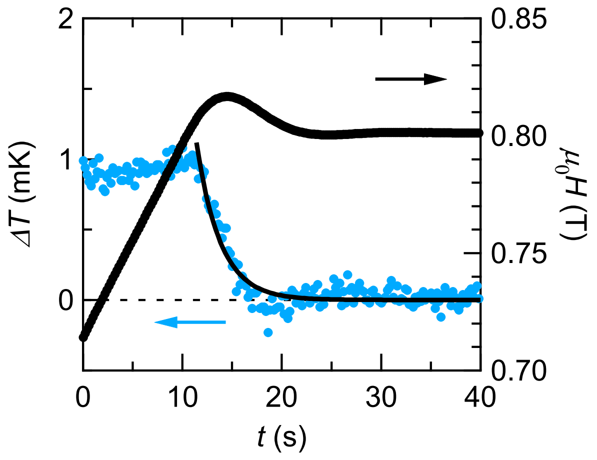

Supplementary Figure 2 | Heating and relaxation processes at T = 0.26 K and $\mu_0 H$ = 0.8 T. Time variation in ΔT [identical to Fig. 1(a)] and the magnetic field determined by the magnet current monitor. A small overshoot of the magnetic field is seen. This overshoot does not enable us to obtain reliable values of the relaxation time shorter than 2 s.

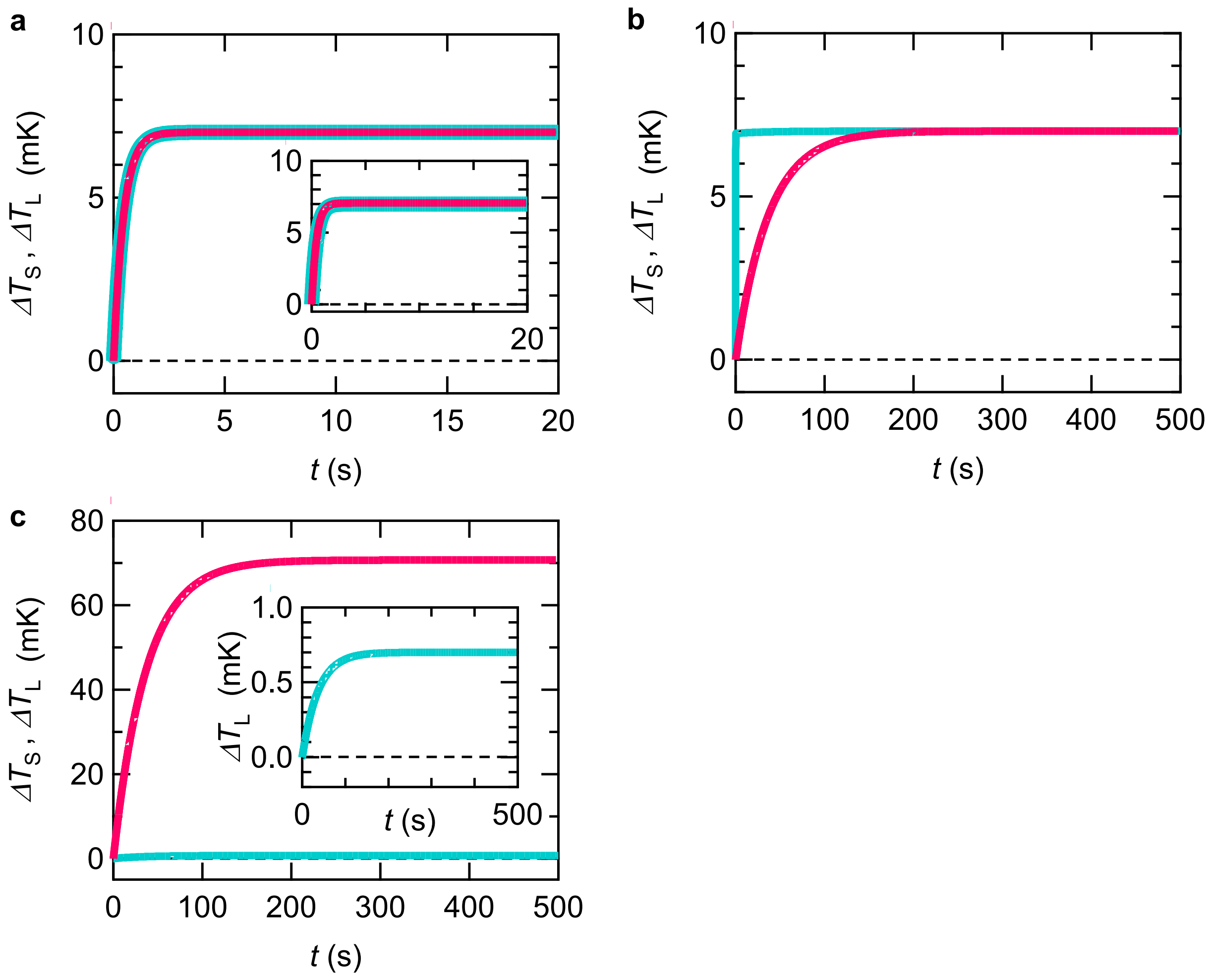

Supplementary Figure 3 | Simulation results of the heating process. (a) Time variation in the temperature elevation of the spins and lattice, ΔT_{S} and ΔT_{L}, for the lattice heating. The spins are strongly coupled to the lattice. Inset: the simulation result for the spin heating. T_{S} is always identical to T_{L} for both the heating methods. The time variation in ΔT_{S} and ΔT_{L} for (b) the lattice and (c) spin heatings, when the spins are decoupled from the lattice. Inset of (c): the enlarged figure of the main panel. Magenta and cyan lines represent ΔT_{S} and ΔT_{L}, respectively.
